# Anti-Allergic Role of Cholinergic Neuronal Pathway via α7 Nicotinic ACh Receptors on Mucosal Mast Cells in a Murine Food Allergy Model

**DOI:** 10.1371/journal.pone.0085888

**Published:** 2014-01-16

**Authors:** Takeshi Yamamoto, Toshihisa Kodama, Jaemin Lee, Naho Utsunomiya, Shusaku Hayashi, Hiroshi Sakamoto, Hirofumi Kuramoto, Makoto Kadowaki

**Affiliations:** 1 Division of Gastrointestinal Pathophysiology, Institute of Natural Medicine, University of Toyama, Toyama, Toyama, Japan; 2 Department of Physical Therapy, Health Science University, Fujikawaguchiko, Yamanashi, Japan; 3 Department of Applied Biology, Kyoto Institute of Technology, Kyoto, Kyoto, Japan; Chiba University Center for Forensic Mental Health, Japan

## Abstract

The prevalence of food allergy (FA) has increased in developed countries over the past few decades. However, no effective drug therapies are currently available. Therefore, we investigated cholinergic anti-inflammatory pathway as a regulatory system to ameliorate disrupted mucosal immune homeostasis in the gut based on the pathophysiological elucidation of mucosal mast cells (MMCs) in a murine FA model. BALB/c mice sensitized with ovalbumin received repeated oral ovalbumin for the development of FA. FA mice developed severe allergic diarrhea and exhibited enhanced type 2 helper T (Th2) cell immune responses in both systemic immunity and mucosal immunity, along with MMCs hyperplasia in the colon. MMCs were localized primarily in the strategic position of the mucosal epithelium. Furthermore, the allergic symptoms did not develop in p85α disrupted phosphoinositide-3 kinase-deficient mice that lacked mast cells in the gut. Vagal stimulation by 2-deoxy-D-glucose and drug treatment with nicotinic ACh receptor (nAChR) agonists (nicotine and α7 nAChR agonist GTS-21) alleviated the allergic symptoms in the FA mice. Nicotine treatment suppressed MMCs hyperplasia, enhanced MPO and upregulated mRNA expression of Th1 and Th2 cytokines in the FA mice colon. MMCs, which are negatively regulated by α7 nAChRs, were often located in close proximity to cholinergic CGRP-immunoreactive nerve fibers in the FA mice colon. The present results reveal that the cholinergic neuroimmune interaction via α7 nAChRs on MMCs is largely involved in maintaining intestinal immune homeostasis and can be a target for a new therapy against mucosal immune diseases with homeostatic disturbances such as FA.

## Introduction

Food allergy (FA) is an abnormal immunologic reaction to food proteins, and its prevalence has increased in developed countries over the past few decades. However, the pathogenic mechanisms underlying FA are not fully understood, and no effective drug therapies are yet available. The only way to avoid allergic reactions to food is to avoid the foods that trigger these responses [1].

It is well known that type 2 helper T (Th2) cell-biased immune responses are responsible for allergic diseases. There is a growing body of work that has examined the interaction between T cells and mast cells and how these relationships might alter immunity to foreign antigens [2]. Th2 cell-associated cytokines, such as IL-4, IL-5, IL-9 and IL-13, are involved in B cell class switching to IgE and in the differentiation and maturation of mast cells and Th2 cells [3], [4], [5].

In the pathological Th2 cell-biased immunological environment, mast cells are usually positioned close to the external environment to respond to the pathogens, and mast cells play a central role as an effector cell and a conductor cell in the pathogenesis of various allergic diseases [5], [6]. The activation of mast cells results in the release of various inflammatory mediators, such as proteases, eicosanoids, biogenic amines, cytokines and chemokines [2]. Despite their strategic location in the mucosa of the intestinal microenvironment, the role of mast cells in FA has not been well investigated.

We previously demonstrated that the number of mucosal mast cells (MMCs) is greatly increased in the colonic mucosa of ovalbumin (OVA)-induced FA mice [7]. Mast cells are classified as MMCs or connective tissue mast cells (CTMCs). Considerable evidence has demonstrated that MMCs are morphologically, biochemically and functionally distinct from CTMCs [2]. CTMCs reside in connective tissues, such as the skin, whereas MMC progenitors migrate to intestinal or bronchial mucosal tissues, where MMCs are able to mature following antigen exposure. CTMCs contain high concentrations of histamine in their granules and mainly contribute to allergic symptoms, such as congestion, itch, urticaria and anaphylaxis, whereas MMCs contain low concentrations of histamine [8]. Consequently, anti-histamine agents have no effect on FA. Furthermore, CTMC stabilizers (e.g., tranilast, ketotifen, and cromolyn) are frequently used for the treatment of various allergic disorders, while these stabilizers fail to exert therapeutic effects on FA, which is supported by our finding that these stabilizers are not able to suppress MMC activation [9].

Recent advances in neuroscience and immunology have revealed a bidirectional interaction between the nervous and immune systems. Therefore, the function of the intestine may be modulated by neuro-immune interactions, but this interaction is not fully understood. Mast cells are ubiquitous in the body, and these cells are often found in close proximity to nerve fibers in various tissues, including the lamina propria of the intestine [3], [4], [10], [11]. Mast cells and nerves are assumed to communicate bilaterally to modulate neuro-physiological effects and mast cell functions [12], which suggests that the neuro-immune interaction may be involved in the pathology of allergic diseases.

Until now, findings on the cholinergic regulation of immune responses have been accumulated in various inflammatory experimental models [13], [14], [15], [16], while there has been little information obtained on the cholinergic control in allergic models, especially in FA models. We have already shown that nicotine exerts an inhibitory effect on antigen-induced degranulation in mucosal-type murine bone marrow-derived mast cells (mBMMCs) through α7 nicotinic ACh receptors (nAChRs), which is reversed by the α7 nAChR antagonist methyllycaconitine [17].

Therefore, an elucidation of the cholinergic regulatory pathway has provided new concepts in understanding and treating FA.

## Materials and Methods

### Ethics statement

This study was carried out in strict accordance with the recommendations in the Guide for the Care and Use of Laboratory Animals of the National Institutes of Health. The Animal Experiment Committee at the University of Toyama approved all of the animal care procedures and experiments (authorization no. S-2009 INM-9).

### Animals

Male BALB/c mice, C57BL/6 mice and BALB/c nude (nu/nu) mice (5 weeks old) were purchased from Japan SLC (Shizuoka, Japan). BALB/c background p85α subunit-disrupted class Ia phosphoinositide-3 kinase (PI3K)-deficient (PI3K^−/−^) mice were gifted from Drs. Shigeo Koyasu (Keio University, Tokyo, Japan) and Takashi Kadowaki (the University of Tokyo, Tokyo, Japan). All mice were housed in the experimental animal facility at the University of Toyama and were provided free access to food and water.

### Induction of food allergy

The murine model of FA was initiated as described previously.8 Briefly, male BALB/c mice (5 weeks old) were sensitized twice at 2-week intervals using an intraperitoneal injection of 50 µg OVA (fraction V; Sigma-Aldrich, St. Louis, MO) in the presence of 1.3 mg/100 µl aluminium hydroxide gel adjuvant (Sigma-Aldrich). Two weeks after systemic priming, the mice received repeated oral administrations of 50 mg/0.3 ml OVA every other day. Diarrhea resulting from FA was assessed by visual monitoring for up to 1 hour following oral challenge. Profuse liquid stool was detected as allergic diarrhea, and the diarrhea-presenting mice were considered to be FA mice.

For the pharmacological evaluation of an activation of nicotinic AChRs (nAChRs) in the FA model, a non-selective nAChR agonist nicotine (Sigma-Aldrich) or a selective α7 nAChR agonist GTS-21 (10 mg/kg, Taiho, Tokushima, Japan) was subcutaneously administered to mice 1 hour before the oral OVA challenges. A central vagal stimulant 2-deoxy-D-glucose (2-DG, 200 mg/kg, Sigma-Aldrich) was intraperitoneally administered 30 minutes before OVA challenges. Furthermore, hexamethonium (C6, 100 mg/kg, Sigma-Aldrich) as a non-selective nAChR antagonist was intraperitoneally administered 30 minutes before 2-DG administration. A CTMC stabilizer disodium cromoglycate (cromolyn, 100 mg/g, Sigma-Aldrich), a CTMC and MMC stabilizer doxantrazole (10 mg/kg, Sigma-Aldrich) or an anti-inflammatory drug prednisolone (10 mg/kg, Sigma-Aldrich) was administered 1 hour before OVA challenges.

One hour after the 6th oral OVA challenge, the proximal colon and spleen were used for morphological and biological analyses, and plasma was for measurement of OVA-specific IgE and mouse mast cell protease (mMCP)-1 (a marker of mouse MMCs) levels. Plasma OVA-specific IgE level was measured using an ELISA kit (DS Pharma Biomedical, Osaka, Japan). Plasma mMCP-1 level was measured using an ELISA kit (Moredun Scientific). These measurements were performed according to the manufacturers' instructions.

### Measurement of myeloperoxidase activity

One hour after the 6th oral OVA challenge, the entire colon of each mouse was excised. Each colon was weighed and cut into small pieces. The colon was homogenized on ice in 1 ml of 0.5% hexadecyltrimethylammonium bromide in 50 mM potassium phosphate buffer (pH 6.0) using a Polytron homogenizer (Kinematica, Littau, Switzerland) and fortified with an additional quantity of the same buffer to 100 mg of the colon in 1 ml. The homogenate was frozen and thawed three times and then was vortex-mixed. The suspension was centrifuged at 15,000×*g* for 20 minutes at 4°C. Myeloperoxidase (MPO) activity of the resulting supernatant was measured. The supernatant (100 µl) was vortex-mixed with 2.87 ml of 0.5 mM *o*-dianisidine in 50 mM potassium phosphate buffer (pH 6.0) and 30 µl of 0.05% hydrogen peroxide. The mixture was measured spectrophotometrically at 460 nm for 4 minutes of incubation. MPO activity is expressed as units per wet weight of the colon in grams (gwwt).

### Expression of cytokine mRNA

One hour after the 6th oral OVA challenge, 2 cm of the proximal colon and spleen were excised. Total RNA was extracted from the tissues using Sepasol Super (Nacalai Tesque, Kyoto, Japan) according to the manufacturer's instructions. Reverse transcription was performed using the Exscript RT reagent kit (Takara Bio, Ohtsu, Japan) and random primers, followed by real-time PCR. Real-time PCR amplification of IL-4, IL-5, IFN-γ and GAPDH was performed using SYBR Premix Ex Taq (Takara Bio). The following primer pairs were used:

IL-4, forward 5′-GGTCTCAACCCCCAGCTAGT-3′ and reverse 5′-GCCGATGATCTCTCTCAAGTGAT-3′;

IL-5, forward 5′-GAAGTGTGGCGAGGAGAGAC-3′ and reverse 5′-GCACAGTTTTGTGGGGTTTT-3′;

IFN-γ, forward 5′-CGGCACAGTCATTGAAAGCCTA-3′ and reverse 5′-GTTGCTGATGGCCTGATTGTC-3′;

GAPDH, forward 5′-TGACCACAGTCCATGCCATC-3′ and reverse 5′-GACGGACACATTGGGGGTAG-3′.

The PCR reaction conditions consisted of 10 seconds at 95°C followed by 40 cycles of 5 seconds at 95°C and 20 seconds at 63°C. Target mRNA levels were normalized to those of GAPDH as an internal control in each sample. The results are expressed as the ratio relative to the naïve group average.

### Morphological analysis

One hour after the 6th oral OVA challenge, the proximal colon was excised. The proximal colon was fixed with 4% paraformaldehyde (w/v) in 0.1 M sodium phosphate buffer (PB; pH 7.3) and immersed for 12–18 hours at 4°C. The tissue was washed with 0.01 M phosphate-buffered saline (PBS; pH 7.3), cryoprotected with 30% sucrose in 0.01 M PBS and embedded in OCT compound. Frozen sections (25 µm) were cut at −20°C using a cryostat microtome (Leica Microsystems, Nussloch, Germany). The sections were soaked for 12–18 hours in 0.01 M PBS containing 0.3% Triton X-100 to increase permeability, and they were then exposed to normal donkey serum (1∶10; Jackson Immunoresearch Laboratories, West Grove, PA) for 30 minutes to reduce the nonspecific binding of antisera and washed in 0.01 M PBS. The sections were exposed to each primary antibody for 12–18 hours, washed with 0.01 M PBS and incubated with the appropriate secondary antibody for 2 hours. All incubations were performed at room temperature. The following primary antibodies were used: goat anti-choline acetyltransferase (ChAT) (1∶200; Chemicon, Temecula, CA), sheep anti-mouse mast cell protease (mMCP)-1 (1∶5,000; Moredun Scientific, Midlothian, UK) and rabbit anti- CGRP (1∶10,000; Chemicon). Cy3-conjugated donkey anti-sheep IgG (1∶200; Jackson Immunoresearch Laboratories, West Grove, PA) and Alexa 488-conjugated donkey anti-rabbit IgG (1∶200; Jackson Immunoresearch Laboratories) were used as secondary antibodies. The preparations were examined using a fluorescence microscope (IX71 system; Olympus, Tokyo, Japan) with a U-MWIG3 filter set (Olympus) and photographed using an Olympus digital camera (DP70; Olympus). The preparations were also observed using confocal laser-scanning microscopy (LSM700; Carl Zeiss, Oberkochen, Germany) to examine the proximity between mMCP-1-positive cells and CGRP-immunoreactive (IR) nerve fibers in the colonic mucosa of the FA mice. Stacks of confocal sections were three-dimensionally reconstructed and observed using Imaris software (Bitplane, Zurich, Switzerland). Surface rendering of each channel was performed in an optimal intensity threshold setting. The intensity threshold setting was optimized by visual comparison between the original and the three-dimensional rendered result.

Furthermore, a segment of the proximal colon was opened along the mesenteric border and fixed with 4% paraformaldehyde. The whole-mount preparation of longitudinal muscle with adherent enteric plexus was dissected from the proximal colon, and the aforementioned immunohistochemical procedures were performed to examine the ChAT and CGRP immunoreactivities in nerve cell bodies of the enteric neurons.

Sections (1 µm thick) of the fixed colon embedded in LR white resin (Sigma-Aldrich) were stained with 1% toluidine blue and observed with light microscopy for the analysis of mast cells.

The brightness and contrast of the images were modified using Adobe Photoshop Elements 2.0 (Adobe Systems, San Jose, CA).

### Electron microscopy

For electron microscopy, the FA mice were perfusion-fixed with 2% paraformaldehyde and 2.5% glutaraldehyde in 0.1 M PB and the proximal colon was removed. The colon was transversely sectioned into 0.5–1.0 mm thick sections with a razor blade. The sections were post-fixed for 1 hour by 1% OsO_4_ in 0.1 M PB, dehydrated in graded ethanol and embedded in EPON 812 (Shell Chemical, Houston, Tex). Ultrathin sections were cut using an LKB 2088-V ultramicrotome (LKB, Bromma, Sweden). The ultrathin sections were collected on copper grids, stained with lead citrate and uranyl acetate and examined with an electron microscope (H-7500, Hitachi, Tokyo, Japan).

### Statistical analysis

The data are expressed as the means ±SE. The statistical comparisons were performed using Student's unpaired *t*-test, the chi-squared test and one-way repeated measures ANOVA followed by Tukey's HSD. A significant difference was defined as *P*<0.05.

## Results

### Induction of food allergy in mice

**Figure 1:**
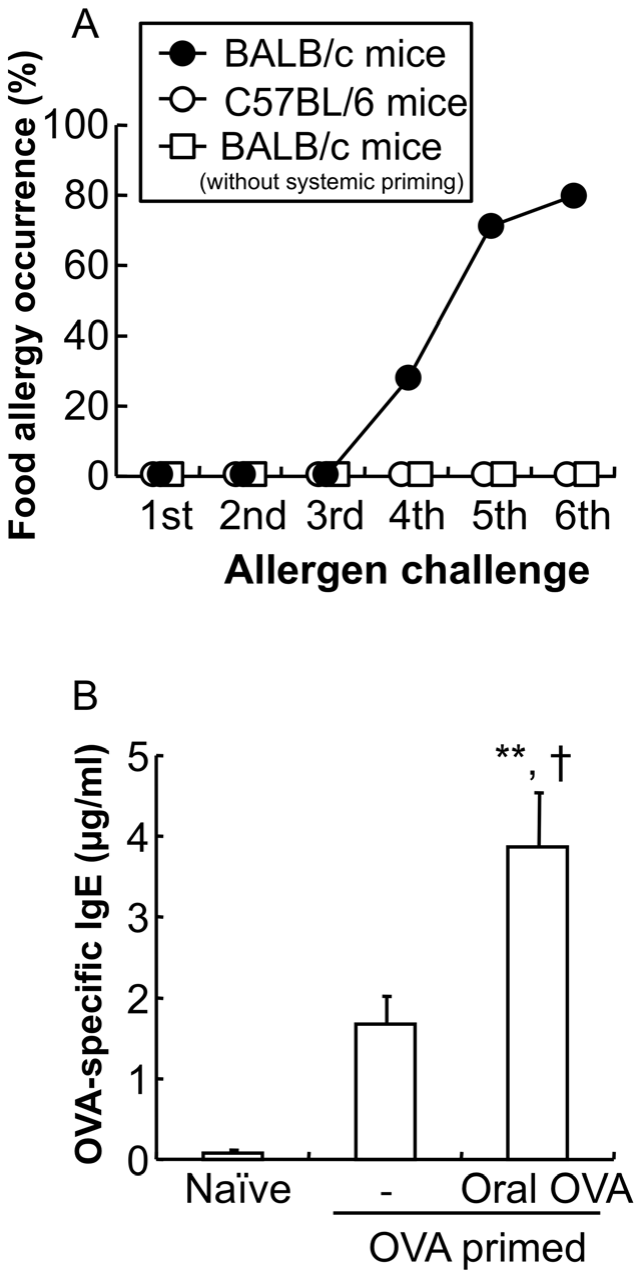
Induction of Th2-mediated food allergy. The allergic responses were induced by two instances of systemic OVA priming and repeated oral OVA (50 mg) challenges. *A*: Systemically primed BALB/c mice (closed circle) and C57BL/6 mice (open circle) and non-systemically primed BALB/c mice (open square) were subjected to food allergy (FA)-inducing oral OVA challenges (*n* = 5–8 mice per group). *B*: The level of OVA-specific IgE in the plasma of naïve mice, systemically primed mice, and systemically primed and repeated oral OVA challenged mice is shown. The level of OVA-specific IgE in the plasma was determined using an ELISA kit. Data are expressed as means ±SE. **P<0.01 vs. naïve mice. †*P*<0.05 vs. systemically primed mice (*n* = 5 mice per group).

Repeated oral OVA challenges to systemically OVA primed mice produced FA associated with allergic diarrhea in Th2-prone BALB/c mice (FA mice, *n* = 5–7), but not in Th1-prone C57BL/6 mice (*n* = 8) (Figure 1A). The occurrence of allergic diarrhea reached about 80.0% after the 6th OVA challenge. However, repeated OVA challenges without systemic OVA priming did not induce allergic diarrhea in BALB/c mice (*n* = 5) (Figure 1A).

The plasma level of OVA-specific IgE was elevated after the systemic OVA priming, and subsequent repeated oral OVA challenges caused a remarkable elevation of the OVA-specific IgE level (naïve mice 0.0±0.0 µg/ml, OVA primed mice 1.7±0.2 µg/ml, oral OVA challenged OVA primed mice 3.8±0.7 µg/ml; **P<0.01 vs. naïve mice, †*P*<0.05 vs. OVA primed mice; *n* = 5 mice per group) (Figure 1B).

### Systemic and mucosal immune responses in food allergy mice

**Figure 2:**
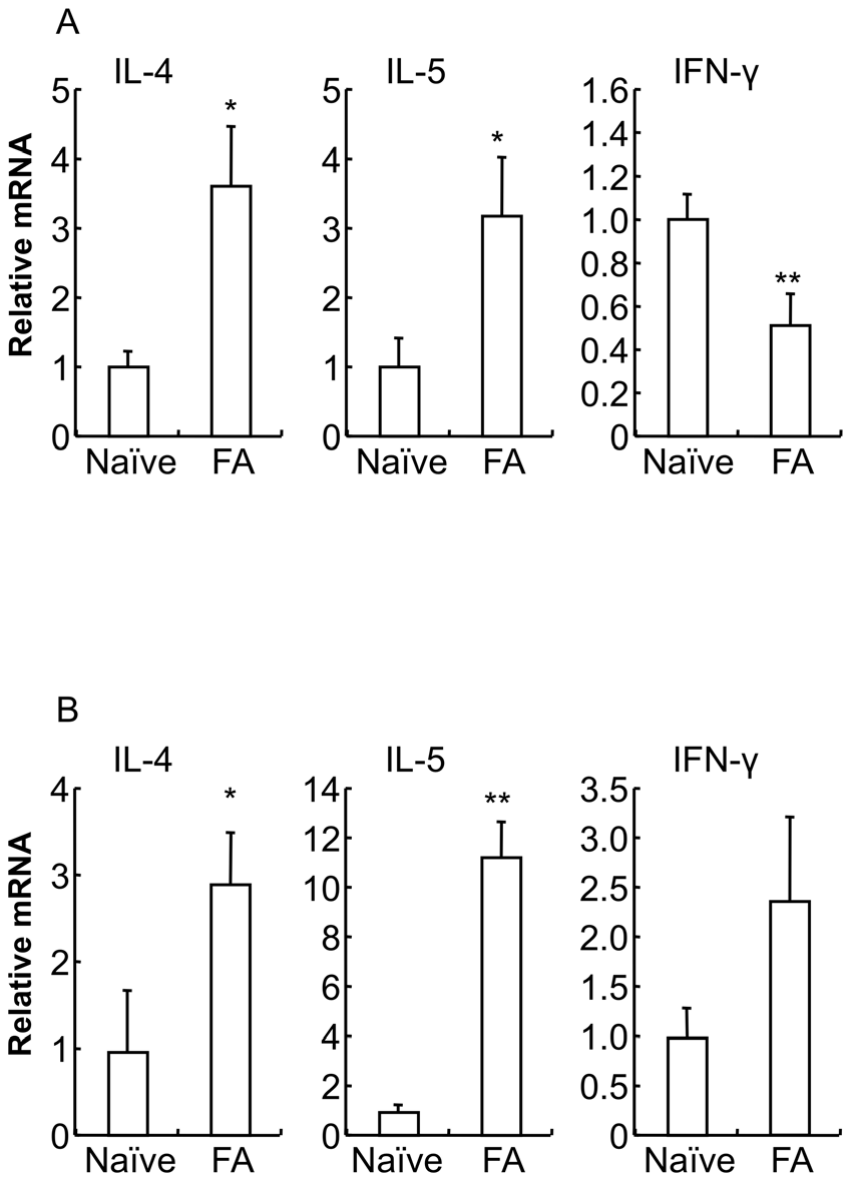
Induction of predominant Th2 cytokine responses in food allergy mice. IL-4, IL-5 and IFN-γ cytokine mRNA expression in the spleen (*A*) and proximal colon (*B*) from naïve mice and FA mice were measured by real-time PCR. Relative mRNA levels of cytokines were normalized to GAPDH expression. Data are expressed as means ±SE. *P<0.05, **P<0.01 vs. naïve mice (*n* = 4–8 mice per group).

The expression of Th1 and Th2 cytokine mRNA was measured using real-time PCR. In systemic immunity (spleen, Figure 2A), mRNA levels of Th2 cytokines (IL-4 and IL-5) were significantly increased in the FA mice (IL-4: naïve mice 1.0±0.2, FA mice 3.7±0.8, *P<0.05 vs. naïve mice; IL-5: naïve mice 1.0±0.4, FA mice 3.2±0.9, *P<0.05 vs. naïve mice; *n* = 5–6 mice per group), whereas the Th1 cytokine (IFN-γ) mRNA level was significantly decreased in the FA mice (naïve mice 1.0±0.1, FA mice 0.5±0.1; **P<0.01 vs. naïve mice; *n* = 6 mice per group). On the other hand, in mucosal immunity (proximal colon, Figure 2B), Th2 cytokine (IL-4 and IL-5) mRNA levels were significantly increased in the FA mice (IL-4: naïve mice 1.0±0.7, FA mice 2.9±0.6, *P<0.05 vs. naïve mice; IL-5: naïve mice 1.0±0.2, FA mice 11.3±1.4, **P<0.01 vs. naïve mice; *n* = 4 mice per group), and the Th1 cytokine (IFN-γ) mRNA level exhibited a tendency to be increased in the FA mice (naïve mice 1.0±0.3, FA mice 2.4±0.8; *n* = 5–8 mice per group).

### Mucosal mast cell hyperplasia in the colon of food allergy mice

**Figure 3:**
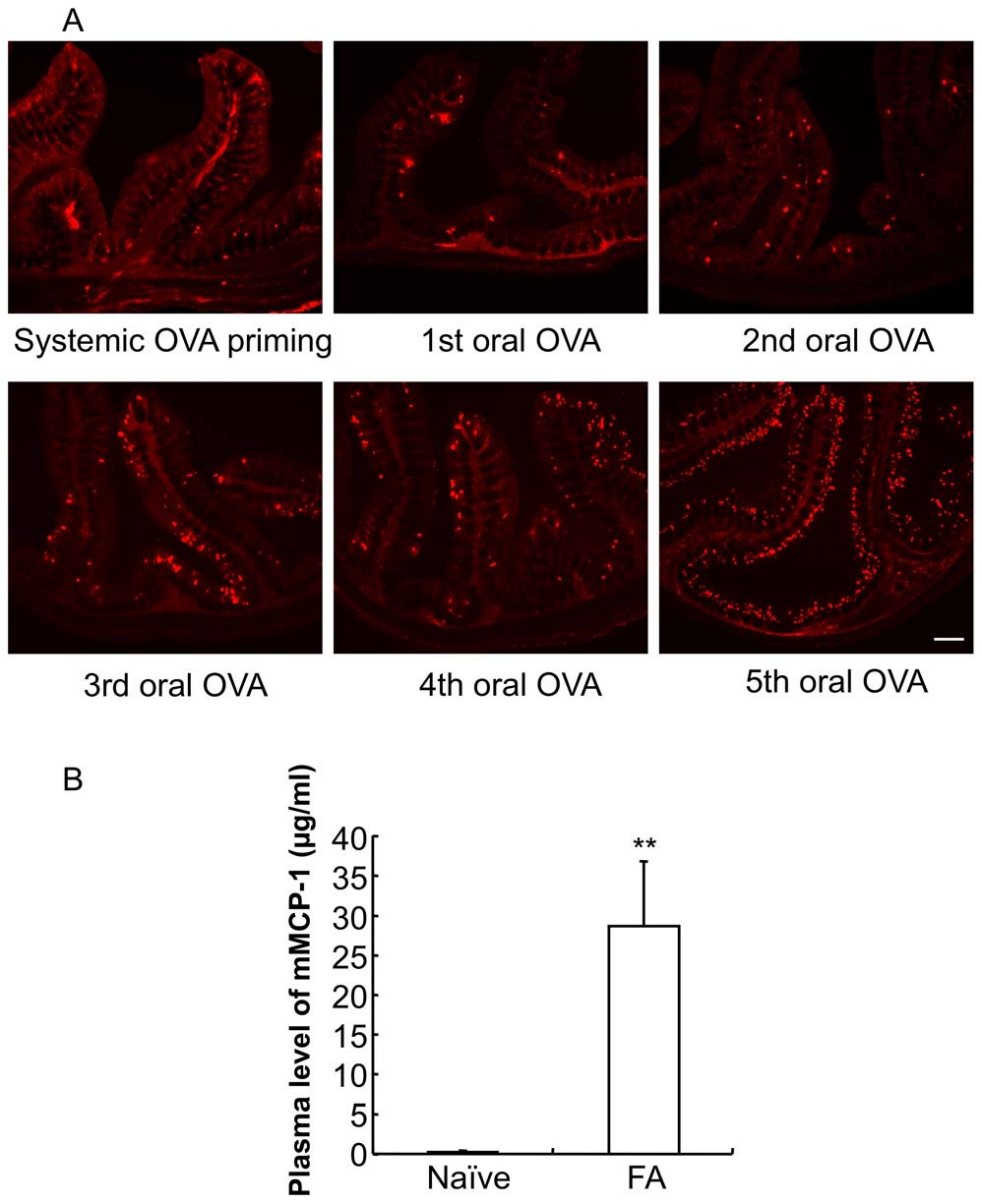
Mucosal mast cell hyperplasia and degranulation in the colon of food allergy mice. *A*: The proximal colons of systemically OVA primed mice and oral OVA-challenged mice after each oral OVA challenge were stained with anti-mouse mast cell protease-1 (mMCP-1) antibodies. Scale bar represents 200 µm. *B*: The concentration of mMCP-1 in the plasma of naïve mice and FA mice is shown. The concentration of mMCP-1 in the plasma was determined using an ELISA kit. Data are expressed as means ±SE. **P<0.01 vs. naïve mice (*n* = 5 mice per group).

We investigated mucosal mast cells in the proximal colon by immunohistochemistry. As shown in Figure 3A, very few MMCs (mMCP-1-positive cells) were scattered in the lamina propria and submucosal layer of the mice colon two weeks after twice systemic OVA priming. The distribution of MMCs was similar to that in the proximal colon of the vehicle-treated control mice. MMCs began to occur in the mucosal epithelium of the primed mice proximal colon two days after the second oral OVA administration, and the number of MMCs was dramatically elevated in the mucosal epithelium of the primed mice colon in proportion to the OVA challenge time. MMCs were localized primarily in the mucosal epithelium of the upper half of the crypts throughout the primed mice colon two days after the fifth oral OVA administration.

To investigate the degranulation of mucosal mast cells, we measured the concentration of plasma mMCP-1 by ELISA (Figure 3B). There was no detectable plasma mMCP-1 in naïve mice. In contrast, the repeated OVA challenges resulted in a dramatic increase in the plasma mMCP-1 level (naïve mice 0.0±0.0 µg/ml, FA mice 29.0±8.0 µg/ml; **P<0.01 vs. naïve mice; *n* = 5 mice per group).

### Involvement of T cell in the development of food allergy in mice

**Figure 4:**
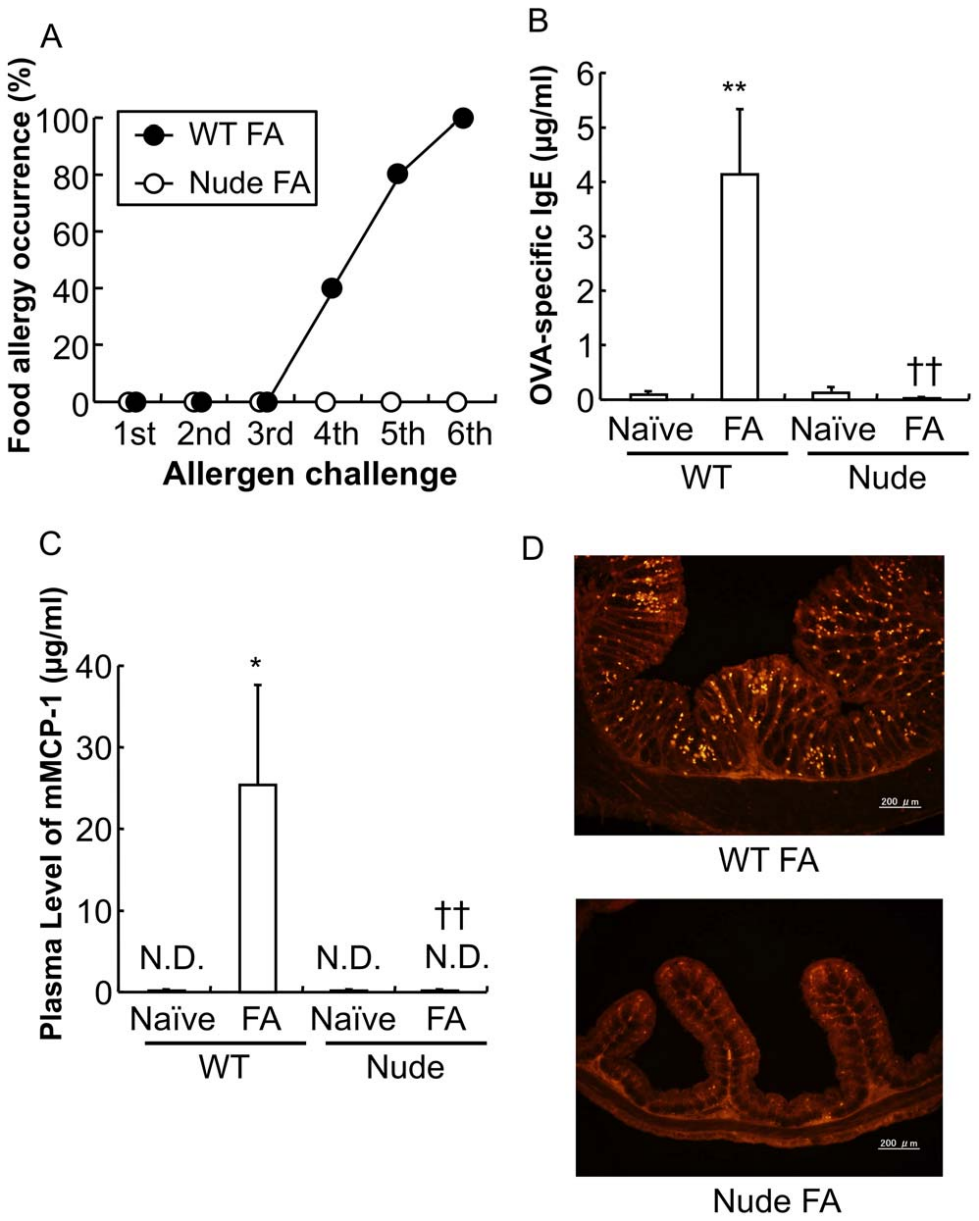
Induction of food allergy in athymic nude mice. *A*: The occurrence of allergic diarrhea in wild-type (WT) FA mice and nude FA mice after each oral OVA challenge is shown (n = 5 mice per group). *B*: The level of OVA-specific IgE in the plasma of WT naïve mice, WT FA mice, nude naïve mice and nude FA mice is shown. The level of OVA-specific IgE in the plasma was determined using an ELISA kit. **P<0.01 vs. WT naïve mice, ††*P*<0.01 vs. WT FA mice (*n* = 3–5 mice per group). *C*: The level of mMCP-1 in the plasma of WT naïve mice, WT FA mice, nude naïve mice and nude FA mice is shown. The level of mMCP-1 in the plasma was determined using an ELISA kit. *P<0.05 vs. WT naïve mice, ††*P*<0.01 vs. WT FA mice (*n* = 3–5 mice per group). *D*: The proximal colon of WT FA mice and nude FA mice after the 6th oral OVA challenge was stained with anti-mMCP-1 antibodies. Scale bars represent 200 µm.

In the FA mouse model, we used BALB/c nude mice that lack functional T cells due to their thymus deficiency. The nude mice did not exhibit any sign of allergic responses following exposure to repeated oral allergen challenges (Figure 4A, *n* = 5 mice per group) and that OVA-specific IgE and mMCP-1 were not detected in the plasma of nude naïve mice and nude FA mice (Figure 4B: **P<0.01 vs. wild-type (WT) naïve mice, ††*P*<0.01 vs. WT FA mice: *n* = 3–5 mice per group; Figure 4C: *P<0.05 vs. WT naïve mice, ††*P*<0.01 vs. WT FA mice: *n* = 3–5 mice per group). Furthermore, no increase of mucosal mast cells was observed in the mucosal layer of the proximal colons of nude FA mice as compared to those of WT mice in the FA model (Figure 4D).

### Characteristics of mast cells in the colon of food allergy mice

**Figure 5:**
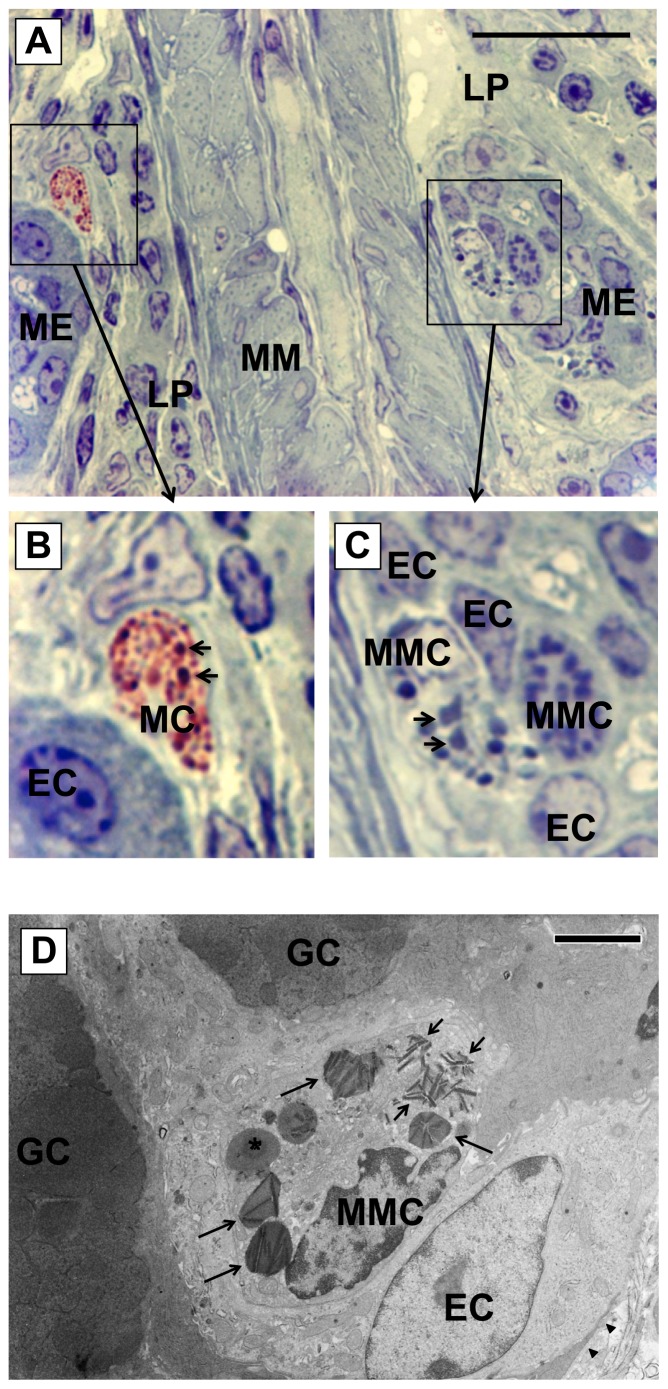
Morphology of mast cells in the colon of food allergy mice. Sections (1 µm thick) of the fixed colon embedded in resin were stained with 1% toluidine blue and were observed with light microscopy for the analysis of mast cells. *A*: On the left hand side of the picture, a mast cell (surrounded by a square) with metachromatic granules was observed in the lamina propria. On the other side, two mast cells (surrounded with another square) with nonmetachromatic granules were localized in the mucosal epithelium. ME: mucosal epithelium, LP: lamina propria, MM: muscularis mucosa. Scale bar represents 50 µm. *B*: An image of higher magnification of the mast cell in the lamina propria. Most of granules of the mast cell exhibited metachromasia, but a few granules (arrows) were nonmetachromatic. MC: mast cell, EC: epithelial cell. *C*: An image of higher magnification of mucosal mast cells (MMCs) in the mucosal epithelium. The granules in MMCs did not exhibit metachromasia and some of the granules were polymorphic in shape (arrows). MMC: mucosal mast cell. *D*: Electron microscopic analysis of mucosal mast cells in the proximal colon of food allergy mice. A variety of morphologies of granules was seen in a MMC: one (asterisk) showed amorphous inner structure, some (long arrows) showed crystals with amorphous matrix and others (short arrows) seemed to be occupied by the crystalline structure. Arrowheads indicate basement membrane. GC: goblet cell. Scale bar represents 4 µm.

Mast cells were observed in the proximal colon of the FA mice and contained many granules intensely stained with toluidine blue (Figure 5A). Only a few mast cells were observed in the lamina propria. These mast cells contained numerous granules in the cytoplasm, most of which exhibited reddish violet metachromasia with toluidine blue (Figure 5B), indicating that these mast cells exhibit properties of CTMCs because in rodents, CTMC granules stain purple, and MMC granules stain blue with toluidine blue [18]. However, a few granules in these mast cells in the lamina propria were nonmetachromatic and were larger than metachromatic granules (Figure 5B; arrows).

Many MMCs were observed in the mucosal epithelium, and the majority of MMC granules were larger (Figure 5C) and were spherical or polymorphic in shape (Figure 5C; arrows). In addition, these MMC granules, which exhibited nonmetachromasia, were strongly stained with toluidine blue, but some were weakly stained.

### Ultrastructural characteristics of mucosal mast cells in food allergy mice

MMCs in the mucosal epithelium of the FA mice proximal colon have been examined with the electron microscope (Figure 5D). Many MMCs were observed within the mucosal epithelium, and they were characterized by the presence of peculiar large granules (0.5–2.5 µm) in the cytoplasm. MMCs had specific granules of varying size, shape and electron-density. Many of the granules (Figure 5D, long arrows) contained crystalline inclusions with cylindrical shape and had irregular contours and showed polymorphic shapes. Other granules seemed to be occupied by crystalline structures (Figure 5D, short arrows). Some of the granules had relatively homogeneous matrix with round or oval contours without the crystalline inclusion (Figure 5D, asterisk).

### Critical involvement of mast cells in the pathogenesis of food allergy in mice

Mice that specifically lack only mast cells have not been reported/generated; two mouse strains, WBB6F1/J-Kit^W^/Kit^W-v^ and C57BL/6-Kit^W-sh^/Kit^W-sh^ are profoundly deficient in mast cells. These mice are most frequently used to analyze the *in vivo* functions of mast cells, but we could not employ these strains to examine the role of mast cells in the induction of FA because the genetic background of these mice is not BALB/c and these mice have other phenotypic abnormalities, such as gastric ulcer and intestinal motility disorder.

**Figure 6:**
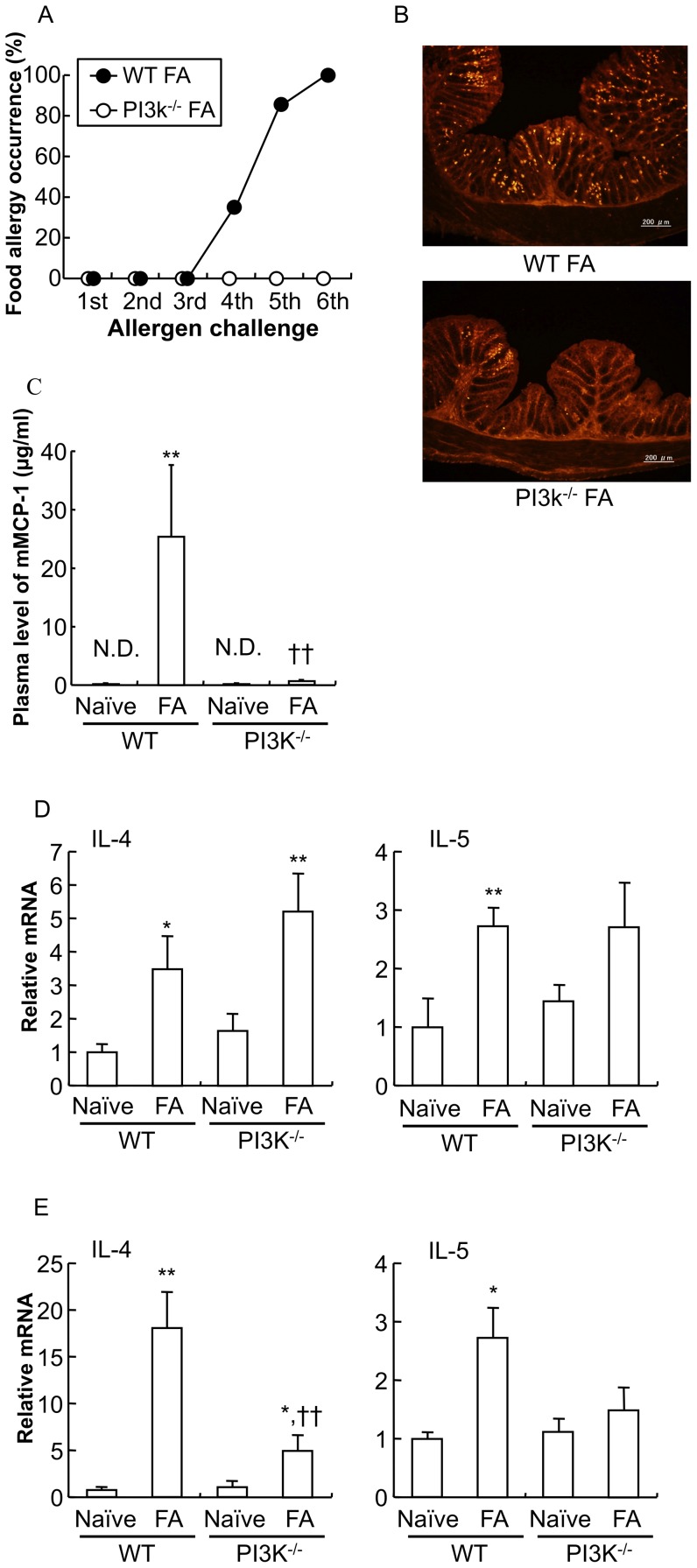
Induction of food allergy in PI3K-deficient mice. PI3K^−/−^ mice as a gastrointestinal mast cell-deficient murine model were subjected to FA-inducing oral OVA challenges. *A*: The occurrence of allergic diarrhea in WT mice (WT FA mice) and PI3K^−/−^ mice (PI3K^−/−^ FA mice) after each oral OVA challenge is shown (*n* = 5 mice per group). *B*: The proximal colon of FA-induced PI3K^−/−^ mice after oral OVA challenge was stained with anti- mMCP-1 antibodies. Scale bars represent 200 µm. *C*: The level of mMCP-1 in the plasma of PI3K^−/−^ naïve mice and PI3K^−/−^ FA mice is shown. The level of mMCP-1 in the plasma was determined using an ELISA kit. Data are expressed as means ±SE. **P<0.01 vs. WT naïve mice, ††*P*<0.01 vs. WT FA mice (*n* = 3–5 mice per group). IL-4 and IL-5 cytokine mRNA expression in the spleen (*D*) and proximal colon (*E*) from WT naïve mice, WT FA mice, PI3K^−/−^ naïve mice and PI3K^−/−^ FA mice were measured by real-time PCR. Relative mRNA levels of cytokines were normalized to GAPDH expression. *P<0.05, **P<0.01 vs. each naïve mice, ††*P*<0.01 vs. WT FA mice (*n* = 4–7 mice per group).

Therefore, in this study, we employed PI3K^−/−^ mice with a BALB/c genetic background that exhibit a selective loss of gastrointestinal mast cells [19]. As a result, PI3K^−/−^ mice failed to develop allergic diarrhea (PI3K^−/−^ FA mice) (Figure 6A, *n* = 5 mice per group). As shown in Figure 6B, immunohistochemical staining with the mMCP-1 antibody revealed that MMCs were dramatically increased in the proximal colon of WT FA mice, whereas only a few mast cells were observed in the mucosal layer of PI3K^−/−^ FA mice. Plasma mMCP-1 was almost undetectable in PI3K^−/−^ naïve mice and PI3K^−/−^ FA mice (Figure 6C: **P<0.01 vs. WT naïve mice, ††*P*<0.01 vs. WT FA mice; *n* = 3–5 mice per group).

Furthermore, in systemic immunity (spleen, Figure 6D), mRNA levels of Th2 cytokines (IL-4 and IL-5) were increased in PI3K^−/−^ FA mice as compared to PI3K^−/−^ naïve mice (IL-4: *P<0.05 vs. WT naïve mice, **P<0.01 vs. PI3K^−/−^ naïve mice; IL-5: **P<0.01 vs. WT naïve mice; *n* = 4–7 mice per group), which is similar to those in WT FA mice. On the other hand, in mucosal immunity (proximal colon, Figure 6E, lower expression of mRNA for Th2 cytokines was found in PI3K^−/−^ FA mice as compared to WT FA mice (IL-4: *P<0.05 vs. WT naïve mice, ††*P*<0.01 vs. WT FA mice; IL-5: *P<0.05 vs. WT naïve mice; *n* = 4–7 mice per group).

### Effect of mast cell stabilizers on the induction of food allergy in mice

**Figure 7:**
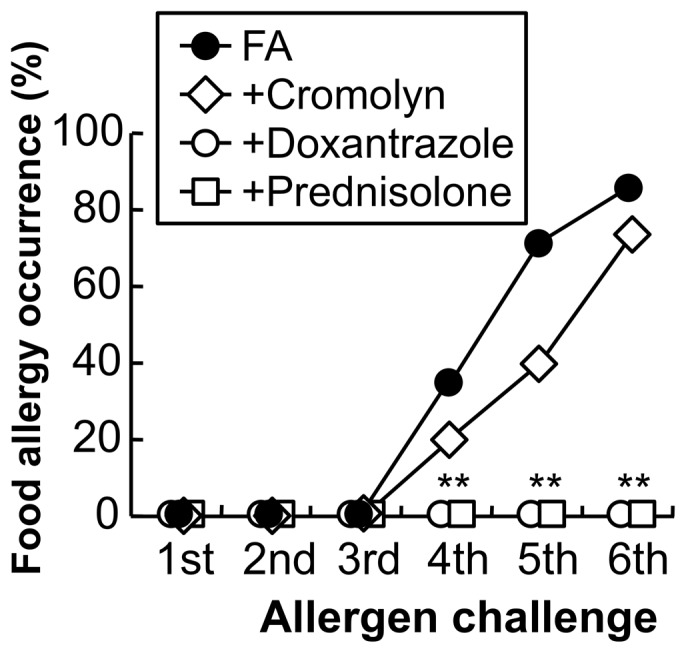
Effect of mast cell stabilizers on the induction of food allergy in mice. The occurrence of allergic diarrhea in FA mice (closed circle) and cromolyn-treated mice (open diamond) after each oral OVA challenge is shown (*n* = 14–15 mice per group). In addition, mice treated with the subcutaneous administration of doxantrazole (open circle, 10 mg/kg) or the oral administration of prednisolone (open square, 10 mg/kg) did not exhibit any sign of allergic responses in the FA model. **P<0.01 vs. FA mice (*n* = 10 mice per each group).

The critical involvement of MMCs in the pathogenesis of FA was pharmacologically investigated in the FA model. The subcutaneous administration of a CTMC and MMC dual stabilizer doxantrazole (10 mg/kg) completely prevented the allergic responses following exposure to repeated oral allergen challenges, whereas the oral treatment with cromolyn (100 mg/kg), a CTMC stabilizer failed to suppress these responses (Figure 7: **P<0.01 vs. FA mice; *n* = 10–15 mice per group). In addition, mice treated with an anti-inflammatory drug prednisolone (10 mg/kg) did not exhibit any sign of allergic diarrhea in the FA model.

### Cholinergic anti-inflammatory pathway in food allergy mice

**Figure 8:**
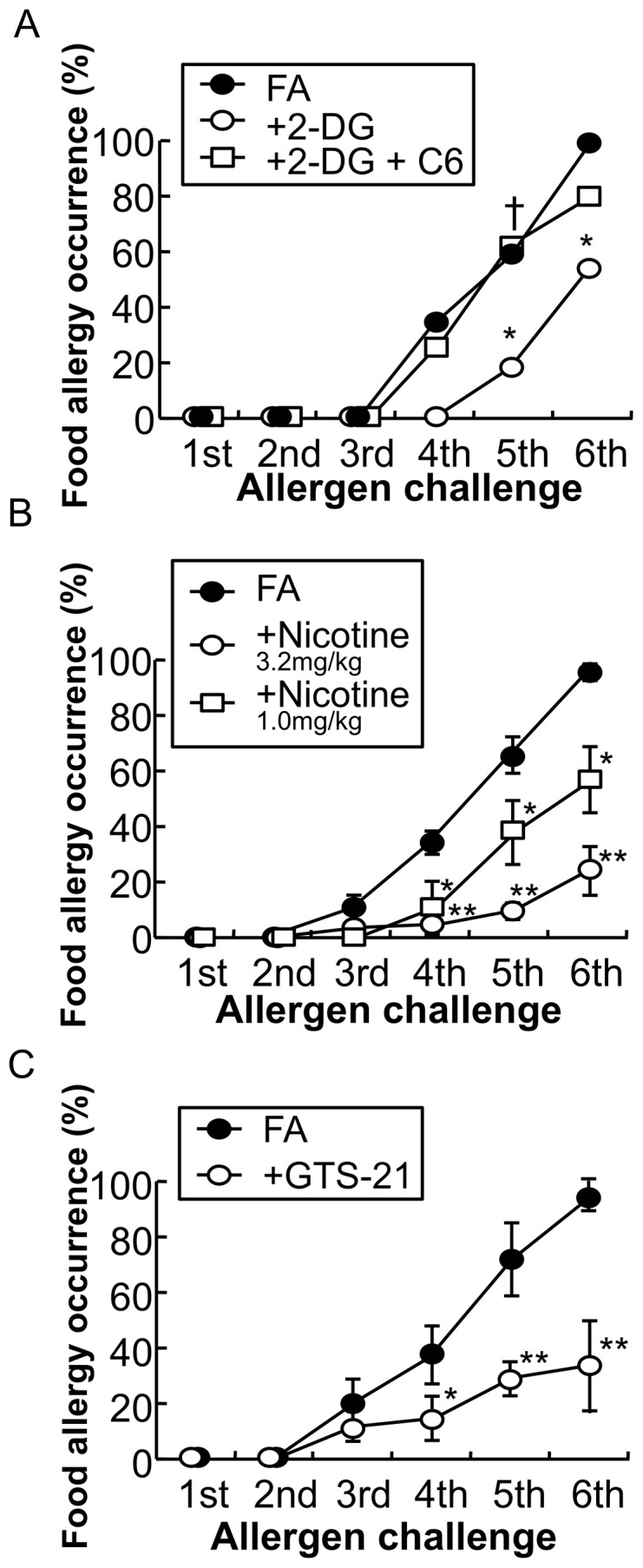
Cholinergic control of food allergy in mice. *A*: The occurrence of allergic diarrhea in FA mice (closed circle), 2-deoxy-d-glucose (2-DG)-treated mice (open circle) and hexamethonium (C6)-treated mice prior to 2-DG administration (open square) after each oral OVA challenge is shown. *P<0.05 vs. FA mice. †*P*<0.05 vs. 2-DG mice (*n* = 11–18 mice per group). *B*: The occurrence of allergic diarrhea in FA mice (closed circle) and nicotine-treated mice (3.2 mg/kg; open circle, 1.0 mg/kg; open square, and 0.32 mg/kg; open diamond) after each oral OVA challenge is shown. *P<0.05, **P<0.01 vs. FA mice (*n* = 10–74 mice per group). *C*: The occurrence of allergic diarrhea in FA mice (closed circle) and GTS-21-treated mice (open circle) after each oral OVA challenge is shown. *P<0.05, **P<0.01 vs. FA mice (*n* = 23–40 mice per group).

To investigate whether vagal activity influences the development of FA, we examined the effect of 2-deoxy-D-glucose (2-DG), a central vagal stimulant on the FA model. Intraperitoneal injection of 2-DG (200 mg/kg) significantly ameliorated allergic diarrhea. After the 6th OVA challenge, the occurrence of allergic diarrhea was significantly decreased from 100.0% to 54.5% (Figure 8A: *P<0.05 vs. vehicle, *n* = 11–18 mice per group). The therapeutic effect of 2-DG was significantly blocked by the subcutaneous injection of the non-selective nAChR antagonist hexamethonium (C6) at 32 mg/kg (Figure 8A: †*P*<0.05 vs. 2-DG, *n* = 11 mice per group).

We next examined the therapeutic effect of nAChR activation on the development of FA. The subcutaneous injection of nicotine attenuated the induction of allergic diarrhea at 1 and 3.2 mg/kg (Figure 8B: *P<0.05, **P<0.01 vs. FA mice, *n* = 10–74 mice per group). The selective α7 nAChR agonist GTS-21 was used to determine whether α7 nAChR mediated the therapeutic effect of nicotine. The subcutaneous injection of GTS-21 (10 mg/kg) resulted in a significant suppression of allergic diarrhea (Figure 8C: *P<0.05, **P<0.01 vs. FA mice, *n* = 23–40 mice per group).

### Effect of nicotine treatment on the systemic and mucosal immune responses in food allergy mice

**Figure 9:**
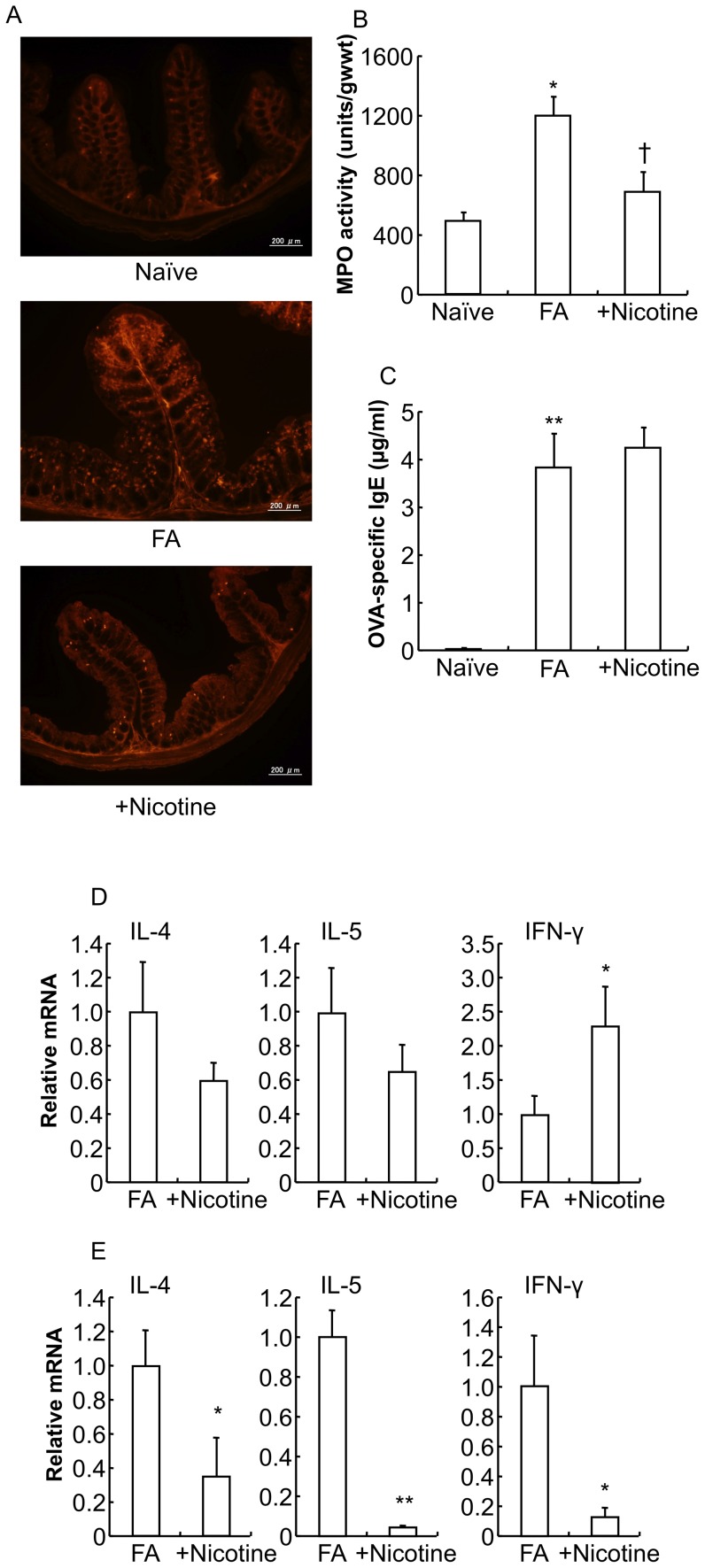
Effect of nicotine on the pathology of food allergy in mice. *A*: The proximal colons of naïve mice, FA mice, and mice treated with the subcutaneous administration of nicotine were stained with mMCP-1 antibodies. Scale bars represent 200 µm. *B*: MPO activity was measured in the colon of naïve mice, FA mice and nicotine-treated FA mice. *P<0.01 vs. naïve mice. †*P*<0.05 vs. FA mice (*n* = 3–10 mice per group). *C*: The level of OVA-specific IgE in the plasma of naïve mice, FA mice and nicotine-treated FA mice is shown. The level of OVA-specific IgE in the plasma was determined using an ELISA kit. **P<0.01 vs. naïve mice (*n* = 5 mice per group). IL-4, IL-5 and IFN-γ cytokine mRNA expression in the spleen (*D*) and proximal colon (*E*) from FA mice and nicotine-treated FA mice were measured by real-time PCR. Relative mRNA levels of cytokines were normalized to GAPDH expression. *P<0.05, **P<0.01 vs. FA mice (*n* = 3–6 mice per group).

The number of MMCs that was dramatically increased in the proximal colon of the FA mice was significantly decreased in that of nicotine-treated FA mice (Figure 9A).

MPO activity in the colon of the FA mice was significantly increased approximately 2.5-fold compared to that in the naïve mice colon, and the subcutaneous injection of nicotine significantly suppressed the increment of MPO activity in the FA mice (Figure 9B: naïve mice 534.8±56.5 units/gwwt, FA mice 1285.7±123.5 units/gwwt, nicotine-treated FA mice 740.5±138.4 units/gwwt; *P<0.05 vs. naïve mice, †*P*<0.05 vs. FA mice; *n* = 3–10 mice per group).

In contrast, the plasma level of OVA-specific IgE remained unchanged in nicotine-treated FA mice compared to the FA mice (Figure 9C: naïve mice 0.1±0.0 µg/ml, FA mice 3.8±0.7 µg/ml, nicotine-treated FA mice 4.3±0.4 µg/ml; **P<0.01 vs. naïve mice; *n* = 5 mice per group).

To investigate the effects of nicotine treatment on the cytokine profile in the FA mice, cytokine mRNA expression was detected by real-time PCR. In systemic immunity (spleen), the upregulated Th2 cytokine (IL-4 and IL-5) mRNA expression observed in the FA mice was not significantly changed by nicotine treatment (Figure 9D: *n* = 6 mice per group), which is almost consistent with no effect of nicotine on the enhanced plasma OVA-specific IgE level in the FA mice (Figure 9C). In contrast, the downregulated Th1 cytokine (IFN-γ) mRNA expression observed in the FA mice was significantly increased in nicotine-treated FA mice (Figure 9D: *P<0.01 vs. FA mice, *n* = 6 mice per group). On the other hand, in mucosal immunity (proximal colon), the upregulated mRNA expression of both Th1 (IFN-γ) and Th2 (IL-4 and IL-5) cytokines was significantly suppressed in nicotine-treated FA mice (Figure 9E: *P<0.05, **P<0.01 vs. FA mice, *n* = 3–6 mice per group).

### Morphological analysis of mucosal mast cell–cholinergic nerve interactions in the colon of food allergy mice

**Figure 10:**
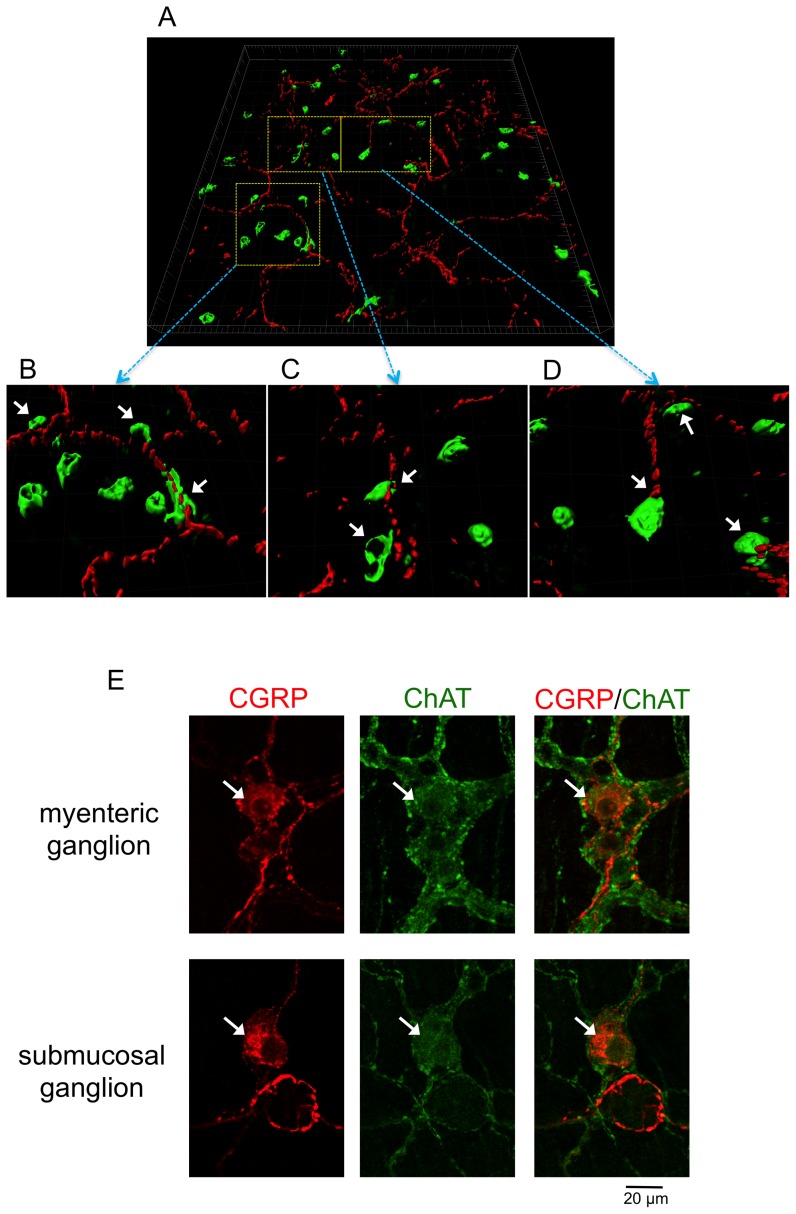
Spatial relationship of mucosal mast cells and cholinergic nerve fibers in the FA mouse colon. *A*: Three-dimensional reconstruction of the confocal images reveals the morphological interaction of mMCP-1-positive MMCs (green) and cholinergic CGRP- immunoreactive nerve fibers (red). Major grid space represents 20 µm. *B*–*D*: High-magnification images of three boxed regions in (*A*). *E*: CGRP- immunoreactive neurons (arrows) exhibited ChAT immunoreactivities in the myenteric and submucosal ganglion of the mouse enteric nervous system. Scale bar represents 20 µm.

We have demonstrated that α7 nAChRs are expressed in mBMMCs and that the stimulation of α7 nAChRs can negatively regulate the activation of mBMMCs [17]. Therefore, we investigated the morphological interaction of MMCs and cholinergic nerve fibers in the lamina propria of the FA mouse proximal colon. It was difficult to investigate the morphological interaction of MMCs and cholinergic nerve fibers using two-dimensional images with fine-resolution in the colon. Therefore, digital images acquired by confocal laser-scanning microscopy were three-dimensionally reconstructed and observed using Imaris software (Figure 10A). The majority of mMCP-1-positive MMCs (green) were located in close vicinity of the CGRP-IR cholinergic nerve fibers (red) in the FA mouse proximal colon (Figure 10B, C, D: arrows). Furthermore, we verified that CGRP-IR neurons (Figure 10E: arrows) exhibited ChAT immunoreactivities in the myenteric and submucosal neurons of the mouse enteric nervous system.

## Discussion

In this study, we provide evidence that MMCs are primarily responsible for the pathology of the FA model and that the cholinergic neuro-immune interaction via α7 nAChRs on MMCs play a crucial role in controlling pathological immune activation to restore the homeostasis in the intestine.

The neuro-immune interaction can be targeted for the design of novel therapeutic agents for the treatment of intestinal immune diseases.

Our murine FA model exhibited various features of type 1 hypersensitive allergic reactions, such as allergic diarrhea within 1 hour after oral antigen exposure, antigen-specific IgE hyperproduction and MMC hyperplasia and degranulation. In addition, Th1-prone C57BL/6 mice did not develop the allergic diarrhea.

Taken together, these data suggest that enhanced Th2-mediated immune responses are responsible for the pathology of our murine FA model.

In the mRNA analysis of cytokines, the FA mice exhibited Th2-biased immune responses in systemic immunity. On the other hand, in mucosal immunity, Th2 immune responses were significantly enhanced, whereas Th1 immune responses were not decreased, but rather increased, indicating that there is a difference between the systemic immune responses and the mucosal immune responses in the FA mice. Furthermore, these enhanced Th2 responses were almost consistent with those observed in other murine OVA-induced FA models, but Th1 responses in the mucosal immunity were different from those in the other models [20], [21]. Kweon et al. observed increased IFN-γ production in the spleen and no IFN-γ production in the colon using ELISA in another murine FA model with allergic diarrhea [21], and Brandt et al. reported no change of IFN-γ mRNA expression in the jejunum of their FA mice with allergic diarrhea [20]. These discrepancies may be due to differences in immunization protocols, cytokine measurement methods and tissue segments. Thus, our murine FA model is characterized by the activation of not only Th2 but also Th1 immune responses in the mucosal immunity of the FA mice colon [7].

Furthermore, to determine whether T cells have a pathological role in the development of FA, we examined whether food allergic diarrhea was induced in nude mice that lack functional T cells due to their thymus deficiency. We found that nude mice failed to exhibit allergic responses in the FA mouse model and that increased OVA-specific IgE and mMCP-1 levels in the plasma and mastocytosis in the mucosa of the proximal colon were not detected in nude naïve mice and nude FA mice. In addition, in this study, OVA-challenged mice without systemic priming failed to exhibit allergic diarrhea. Thus, these results indicate that the T-cell-mediated immune responses in the systemic immunity are essentially required for the development of the allergic responses in our FA model.

This study revealed that mMCP-1 immunoreactive MMCs increased in the mucosal epithelium of the FA mice colon, which is similar to previous reports on the appearance of mast cells in the jejunal mucosa of oral allergen–induced diarrhea mice [20] and parasite-infected mice [22], [23]. Thus, these findings suggest that MMCs are localized in the strategic position to enhance a rapid expulsion of food antigens or parasites from the gut by bioactive products released from MMCs.

The toluidine blue staining revealed a difference between mast cells in the lamina propria and MMCs in the mucosal epithelium of the FA mice colon. Most of the granules of mast cells in the lamina propria, but not MMCs exhibited metachromasia presumably caused by sulfate radicals of proteoglycan such as heparin, suggesting that the difference results from chemical properties of substances contained in the granules of mast cells and that the granules of MMCs in the mucosal epithelium have depleted the reactive substances.

In the previous studies with mMCP-1-deficient mice in the parasite infection model, the light microscopic analysis reveals that MMCs in the jejunal mucosa of the deficient mice have smaller, less intensely toluidine blue-positive granules than those in WT mice and that the granules of MMCs in the deficient mice are uniformly round, whereas those in WT mice are irregularly shaped [23]. Furthermore, previous ultrastructural analysis demonstrates that crystalline inclusions with a cylindrical shape abundantly exist in the irregularly shaped granules of MMCs in the jejunal mucosa of the murine parasite infection model [23], which is consistent with the present findings using the electron microscopy in the granules of MMCs in the mucosal epithelium of the FA mice colon. However, in the mMCP-1 deficient mice, the shapes of MMC granules are regular and oval, and no crystalline structures are observed within the granules in the parasite infection model [23]. Taken all together, it is suggested that the disappearance of matrix constituents such as chondroitin sulfate in the granules of mast cells may be related to the formation of crystalline structures of chymases such as mMCP-1.

The PI3K family is involved in signal transduction that regulates cell proliferation, survival and differentiation. In particular, PI3K is involved in many functions of immune cells [24], and in mast cells, the activation of class Ia PI3K is required in the downstream signaling cascades initiated by the stimulation of high-affinity IgE receptor FcεRI. PI3K^−/−^ mice are observed to be deficient in gastrointestinal mast cells but still have abundant dermal mast cells [19], indicating that the p85α subunit of PI3K is required for the development of gastrointestinal mast cells, but not dermal mast cells.

PI3K^−/−^ mice failed to develop FA in our FA model, which were attributed to a deficiency of mast cells in the intestine, because: 1) mast cells can promote T-cell migration by producing chemokines [25], [26], 2) mast cell products can promote dendritic cells to acquire a Th2 cell-inducing phenotype [2], [27], and 3) mast cells themselves can produce Th2 cytokines, such as IL-4, 5 and 13 [2], [25].

Furthermore, despite our finding that nicotine treatment atteuated allergic diarrhea in the FA mouse model, nicotine had no effect on the enhanced plasma IgE level.

Thus, these data suggest that the pathogenesis of FA is mainly attributed to mast cell hyperplasia in the intestine, but not antigen-specific IgE hyperproduction. Taken all together, our results indicate that MMCs critically contribute to the development of FA through orchestrating Th2 immune responses in the mucosal immunity of the FA mice.

The cross-talk between the autonomic nervous system and immune system is becoming increasingly elucidated to be of great importance for the maintenance of immune homeostasis. Several lines of evidence indicate that parasympathetic vagus nerve, through release of acetylcholine, plays a counter-regulatory role to restore immune homeostasis in diverse inflammatory disease models, such as ischemia-reperfusion injury [28], pancreatitis [29], peritonitis [30], DSS-induced colitis [31] and postoperative ileus [32], [33]. The anti-inflammatory properties of the vagus nerve in these mouse models are primarily attributed to the suppression of macrophage activation that leads to the induction of inflammation through the release of the central inflammatory mediator TNF-α. Recently, it has become clear that nAChRs are expressed in various immune cells [14], [32] as well as macrophages [34]. However, it still remains unclear whether the stimulation of nAChRs on these immune cells ameliorates diverse inflammatory diseases. In particular, it is worth noting that it is unknown whether cholinergic activation via nAChRs serves as an anti- inflammatory pathway in Th2-related diseases, especially allergic diseases.

In this FA study, vagal stimulation with 2-DG ameliorated the development of FA, which was reversed by the nAChR antagonist C6, indicating that vagus nerves exhibit anti-allergic properties via nAChRs. The therapeutic potential of the cholinergic agonist nicotine in inflammatory diseases has recently been demonstrated. In fact, nicotine has been shown to suppress inflammatory responses in various clinical studies, animal models and in vitro studies [12], [35], [36], [37]. Notably, in a randomized double-blind study, nicotine patches in combination with conventional therapy have ameliorated ulcerative colitis, in which Th2-mediated phenomena tend to predominate, as compared to placebo patches and conventional therapy; however, nicotine patch treatment has caused adverse side effects, such as nausea, dizziness, headache and addiction due to non-selectivity of nicotine for subtypes of nAChRs [38].

In this study, nicotine treatment significantly ameliorated FA, mainly due to the suppression of upregulated mucosal immune responses via α7 nAChRs on immune cells. Therefore, the therapeutic effects of nicotine and GTS-21 on the FA model raise the possibility that a strategy for drug discovery against FA by targeting α7 nAChRs could potentially have therapeutic benefits.

Moreover, we have demonstrated that mBMMCs express α7 nAChRs and that their activation exerts inhibitory effects on antigen-IgE-mediated degranulation in mBMMCs [17]. In addition, our results in this study reveal that MMCs can orchestrate the allergic and inflammatory responses in the mucosal immune environment of the FA mice colon. Therefore, it is suggested that nicotine treatment suppresses the activation of MMCs through α7 nAChRs on MMCs, thereby reducing the number of MMCs and the plasma mMPC-1 level, which results in the significant amelioration of FA.

Furthermore, to examine whether the cholinergic anti-allergic pathway is a hard-wired connection between the immune and nervous systems closely interacting to regulate FA, we undertook morphological analysis in the mucosa of the FA mouse colon. Previous reports and this study demonstrate that in the mouse enteric nervous system, CGRP is exclusively contained in intrinsic primary afferent neurons that are immunoreactive for choline acetyltransferase [39], [40], indicating that CGRP-IR neurons contain ACh as a neurotransmitter in the mouse colon. Our morphological findings of the close proximity between MMCs and CGRP-IR cholinergic nerve fibers in the colonic mucosa of the FA mice imply that the neural cholinergic regulation of immune responses occurs via nAChRs on immune cells, which is consistent with previous reports [14], [32]. Collectively, it is suggested that cholinergic nerve fibers closely communicate with MMCs to regulate mast cell functions in the FA mice colon.

Further investigation of this cholinergic counter-regulatory nerve-immune interactions in the intestine will provide new insights to elucidate FA. In addition, new strategies for drug discovery that target the hard-wired connection via α7 nAChRs can lead to novel drug therapies against diverse intestinal immune diseases.

## Supporting Information

Figure S1 **α7 nAChR-mediated control of food allergy in mice.** The occurrence of allergic diarrhea in FA mice (closed circle), GTS-21-treated mice (open circle; 10 mg/kg, p.o.) and MLA-treated mice prior to GTS-21 administration (open square; 1 mg/kg, s.c.) after each oral OVA challenge is shown. *P<0.05, **P<0.01 vs. FA mice. †*P*<0.05 vs. GST-21-treated mice (*n* = 6–18 mice per group).
(DOCX)
