# Supplementary material for: Anti-Allergic Role of Cholinergic Neuronal Pathway via α7 Nicotinic ACh Receptors on Mucosal Mast Cells in a Murine Food Allergy Model
Source: PLoS One. 2014 Jan 16;9(1):e85888. doi: 10.1371/journal.pone.0085888 (PMC3894205; doi:10.1371/journal.pone.0085888)
Supplement: Figure S1 — α7 nAChR-mediated control of food allergy in mice. The occurrence of allergic diarrhea in FA mice (closed circle), GTS-21-treated mice (open circle; 10 mg/kg, p.o.) and MLA-treated mice prior to GTS-21 administration (open square; 1 mg/kg, s.c.) after each oral OVA challenge is shown. *P<0.05, **P<0.01 vs. FA mice. †P<0.05 vs. GST-21-treated mice (n = 6–18 mice per group). (DOCX) [file pone.0085888.s001.docx]

**Supporting Information**

**Figure S1. α7 nAChR-mediated control of food allergy in mice.**

The occurrence of allergic diarrhea in FA mice (closed circle), GTS-21-treated mice (open circle; 10 mg/kg, p.o.) and MLA-treated mice prior to GTS-21 administration (open square; 1 mg/kg, s.c.) after each oral OVA challenge is shown. **P* < 0.05, ***P* < 0.01 vs. FA mice. †*P* < 0.05 vs. GST-21-treated mice (*n* = 6-18 mice per group).
